# Comparative transcriptome and mutation analyses of the pancreatic islets of a rat model of obese type 2 diabetes identifies a frequently distributed nonsense mutation in the lipocalin 2 gene

**DOI:** 10.1093/dnares/dsaf004

**Published:** 2025-02-26

**Authors:** Norihide Yokoi, Naoki Adachi, Tomoki Hirokoji, Kenta Nakano, Minako Yoshihara, Saki Shigenaka, Ryuya Urakawa, Yukio Taniguchi, Yusaku Yoshida, Shigeo Yokose, Mikita Suyama, Tadashi Okamura

**Affiliations:** Laboratory of Animal Breeding and Genetics, Division of Applied Biosciences, Kyoto University Graduate School of Agriculture, Sakyo-ku, Kyoto 606-8502, Japan; Laboratory of Animal Breeding and Genetics, Division of Applied Biosciences, Kyoto University Graduate School of Agriculture, Sakyo-ku, Kyoto 606-8502, Japan; Laboratory of Animal Breeding and Genetics, Division of Applied Biosciences, Kyoto University Graduate School of Agriculture, Sakyo-ku, Kyoto 606-8502, Japan; Department of Laboratory Animal Medicine, Research Institute, National Center for Global Health and Medicine, Shinjuku-ku, Tokyo 162-8655, Japan; Division of Bioinformatics, Medical Institute of Bioregulation, Kyushu University, Higashi-ku, Fukuoka 812-8582, Japan; Laboratory of Animal Breeding and Genetics, Division of Applied Biosciences, Kyoto University Graduate School of Agriculture, Sakyo-ku, Kyoto 606-8502, Japan; Laboratory of Animal Breeding and Genetics, Division of Applied Biosciences, Kyoto University Graduate School of Agriculture, Sakyo-ku, Kyoto 606-8502, Japan; Laboratory of Animal Breeding and Genetics, Division of Applied Biosciences, Kyoto University Graduate School of Agriculture, Sakyo-ku, Kyoto 606-8502, Japan; Biotechnical Center, Japan SLC, Inc., Hamamatsu, Shizuoka 433-8114, Japan; Biotechnical Center, Japan SLC, Inc., Hamamatsu, Shizuoka 433-8114, Japan; Division of Bioinformatics, Medical Institute of Bioregulation, Kyushu University, Higashi-ku, Fukuoka 812-8582, Japan; Department of Laboratory Animal Medicine, Research Institute, National Center for Global Health and Medicine, Shinjuku-ku, Tokyo 162-8655, Japan

**Keywords:** lipocalin 2, nonsense mutation, pancreatic islets, transcriptome, type 2 diabetes

## Abstract

Type 2 diabetes (T2D) is a multifactorial disease caused by insulin resistance and impaired insulin secretion from pancreatic β-cells, but the precise mechanisms remain to be elucidated. To identify primary genetic factors of T2D in a rat model, we performed comparative transcriptome and mutation analyses of the pancreatic islets between the obese Zucker fatty rat and the Zucker fatty rat-derived T2D model Zucker fatty diabetes mellitus (ZFDM) rat. Among differentially expressed genes irrespective of obesity and glucose intolerance states, we identified a nonsense mutation, c.409C > T (p.Gln137X), in the lipocalin 2 (*Lcn2*) gene which encodes a secreted protein called neutrophil gelatinase-associated lipocalin, a well-known biomarker for inflammation. We examined the relevance of the *Lcn2* mutation with T2D in the ZFDM rat by using genome editing and genetic linkage analysis and confirmed that the *Lcn2* mutation exhibits no significant association with the onset of T2D. Interestingly, we found that the *Lcn2* mutation is distributed widely in rat species, such as commonly used DA and F344 strains. Our data indicate that several rat strains would serve as *Lcn2* deficient models, contributing to elucidate the pathophysiological roles of Lcn2 in a wide variety of phenotypes.

## Introduction

Type 2 diabetes (T2D) is a multifactorial disease caused by insulin resistance and impaired insulin secretion from pancreatic β-cells; β-cell failure is a central element in the development and progression of T2D. The β-cell failure in T2D is a complex process involving multiple factors, such as insulin resistance and β-cell overload, lipotoxicity, chronic inflammation, oxidative stress, endoplasmic reticulum stress, and genetic and epigenetic factors. However, the precise mechanisms remain to be elucidated.

Transcriptome analysis of the pancreatic islets offers a powerful technique not only to reveal the pathophysiological state of β-cells but also to identify candidate genes in T2D. There have been several studies to identify candidate genes for T2D by transcriptome analyses in pancreatic islets from individuals with T2D and nondiabetic controls. *PAX5* has been reported as a potential transcriptional regulator of many T2D-associated differentially expressed genes (DEGs) in human islets.^1^ Transcriptome profiling in db/db mice revealed that a reduction in the expression of *Glut2, Ins1, Ins2, MafA, Mt1*, and *Pdx1* was indicative of dedifferentiation in db/db islets.^2^ The Zucker diabetic fatty (ZDF) rat, a model of obese T2D, carries a genetic defect in β-cell gene transcription, in which insulin promoter activity and insulin gene expression were reduced even in lean animals.^3^ They concluded that the genetic reduction in β-cell gene transcription in ZDF rats likely contributes to the development of diabetes in the setting of insulin resistance. Transcriptome profiling of ZDF rats showed an increase in the genes encoding proteases and extracellular matrix components that are associated with tissue remodelling and fibrosis.^4^ In addition, ZDF rats showed an increase in vascular endothelial growth factor-A and Thrombospondin-1 genes, suggesting that an inability of the islet to maintain vascular integrity may contribute to β-cell failure.^5^ However, the primary genetic factors have not been clarified yet.

Among animal models of T2D, the Zucker fatty diabetes mellitus (ZFDM) rat has been derived from the obese Zucker fatty (ZF) rat harbouring a missense mutation (*fatty, fa*) in the leptin receptor (*Lepr*) gene.^6^ Animals homozygous for the *fatty* mutation in both ZF and ZFDM strains exhibit obesity, whereas only male ZFDM rats develop T2D accompanied with histopathological changes in the pancreatic islets such as loss of islet structure and β-cell destruction.^6,7^ In spite of the same origin, there is a significant difference in genetic profiles between the 2 strains^8^; genetic factors involved in the development of T2D remain unknown.

We have previously performed transcriptome analysis of the pancreatic islets in ZFDM rats to examine the mechanism underlying functional differences between non-large and enlarged islets. Together with the insulin secretion experiment and metabolome analysis, we found that enlarged pancreatic islets show tumour cell-like metabolic features of glucose metabolism accompanied with reduced β-cell specific gene expressions and glutamate production, which could contribute to the development of incretin unresponsiveness in obese T2D.^9^ However, the pathogenesis of dysfunction of the pancreatic islets and genetic factors involved in the development of T2D still need to be clarified.

In the present study, to elucidate the gene expression profile of the pancreatic islets and primary genetic factors of T2D in the ZFDM rat, we performed comparative transcriptome and mutation analyses on the pancreatic islets between ZF and ZFDM rats. Among DEGs, we identified a nonsense mutation in a strong candidate gene for T2D, which is found to be existed frequently in rat species. We also evaluated the relevance of the mutation with T2D by both genome editing and genetic linkage analysis.

## Materials and methods

### Ethics approval and consent to participate

All animal experiments were approved by the Committee on Animal Experimentation of Kobe University and Kyoto University and carried out in accordance with the Guidelines for Animal Experimentation at Kobe University and Kyoto University.

### Animals

For isolation of the pancreatic islets and genetic linkage analysis, male ZF rats (Slc:Zucker-*Lepr*^*fa/fa*^ and -*Lepr*^*+/+*^) were purchased from Japan SLC, Inc. and male ZFDM rats (Hos:ZFDM-*Lepr*^*fa/fa*^ and -*Lepr*^*fa/+*^) were provided by Hoshino Laboratory Animals, Inc. All animals were maintained under specific pathogen-free conditions with a 12 h light-dark cycle and were provided with a commercial diet CE-2 (CLEA Japan, Inc.) at the Animal Facility of Kobe Biotechnology Research and Human Resource Development Center of Kobe University. At the end of the experiments, animals were sacrificed by overdose of anaesthesia with pentobarbital sodium (2018 or before).

For genome editing experiment, female and male SD rats (Slc:SD) were purchased from Japan SLC, Inc. and female ZFDM rats (Hos:ZFDM-*Lepr*^*fa/+*^) and male ZFDM rats (Hos:ZFDM-*Lepr*^*fa/fa*^ and -*Lepr*^*fa/+*^) were provided by Hoshino Laboratory Animals, Inc. All animals were maintained under specific pathogen-free conditions with a 14 h-light and 10 h-dark cycle and were provided with a commercial diet F-2 (Oriental Yeast Co., Ltd.) at the Institute of Laboratory Animals, Graduate School of Medicine, Kyoto University. At the end of the experiments, animals were sacrificed by carbon dioxide inhalation (2019 or later).

### Isolation of the pancreatic islets

Pancreatic islets were isolated by the collagenase digestion and Ficoll gradient method.^10,11^ Isolated pancreatic islets were cultured for 3 days in RPMI1640 (Sigma-Aldrich) before experiments.

### RNA sequencing and data analysis

Total RNA was extracted from the pancreatic islets using RNeasy Micro kit (Qiagen). RNA sequencing (125 bp paired-end) was performed on 1 μg each of total RNA, using an Illumina HiSeq 2500 system by Eurofins Genomics. Sequence reads were cleaned using trimmomatic (ver. 0.39),^12^ the quality were checked using FastQC (version 0.11.9),^13^ and then aligned to the rat genome (mRatBN7.2) using STAR (version 2.7.10).^14^ Data were transformed into BAM format using Samtools (version 1.15.1),^15^ and raw read counts were calculated using featureCounts (version 2.0.3).^16^ The following analyses were performed using R (version 4.1.3) (https://www.r-project.org/). Filtering low expression genes, TMM (trimmed mean of M values) normalization, and extraction of DEGs were done using edgeR (version 3.40.1).^17,18^ DEGs were extracted using a quasi-likelihood F-test of edgeR with a threshold of FDR (false discovery rate) < 0.01 and fold change > 2. Information of DEGs was obtained using biomaRt (version 2.54.0).^19,20^ Gene Ontology (GO) and pathway analyses were performed using Database for Annotation, Visualization, and Integrated Discovery (DAVID).^21,22^ GO analysis was also performed using Metascape.^23^ The RNA sequencing data have been deposited in DDBJ Sequence Read Archive (DRA) with the accession numbers DRA007109,^9^ DRA007371,^9^ and DRA012479.

### Mutation analysis using RNA sequencing data

Single nucleotide polymorphisms (SNPs) between ZF and ZFDM rats were detected from BAM files of RNA sequencing data of the pancreatic islets at 8 weeks of age using the Genome Analysis Toolkit (GATK) (version 4.2.6.1)^24^ with the GATK Best Practice.^25,26^ Resulting Variant Calling Format (VCF) files were summarized using VCFtools (version 0.1.16).^27^ Variants were annotated using Variant Effect Predictor (VEP) (version 108).^28^ SNPs were defined as those fixed for distinct homozygous states between ZF and ZFDM rats.

### Mutation screening of the lipocalin 2 gene

Genome DNA was extracted from tail tip samples using the Wizard SV Genomic DNA Purification System (Promega). All amino acids coding regions in the lipocalin 2 gene of ZF and ZFDM rats were sequenced by the Sanger method. A polymerase chain reaction (PCR)-restriction fragment length polymorphism (RFLP) system was developed for genotyping of the Q137X nonsense mutation found in ZFDM rats: PCR product (669 bp) amplified by using a primer set (FW, 5′-aaccctgggtatgacctgaa-3′; RV, 5′-ctggggcctggattattgta-3′) is digested by restriction enzyme *Xsp*I (CTAG), resulting in 464 bp, 114 bp, and 91 bp fragments in ZF rats (wildtype allele) while the 464 bp fragment is further divided into 347 bp and 117 bp fragments in ZFDM rats (mutant allele). The PCR-RFLP system was also applied for genotyping of the mutation among rat species. The genome DNA of 157 inbred rat strains (Supplementary Table S13) were provided by the National Bioresource Project-Rat (NBRP-Rat), Kyoto University (Kyoto, Japan). The genome DNA of the SDT/Jcl rat has been obtained previously.^29^ The ZDF-*Lepr*^*fa*^/CrlCrlj rat was purchased from the Jackson Laboratory Japan, Inc. and genome DNA was extracted.

### CRISPR/Cas9-mediated genome editing in ZFDM rats

*Lcn2* knock-in ZFDM rats, in which the Q137X nonsense mutation was replaced with a wild-type nucleotide, were generated by CRISPR /Cas9-mediated genome editing as described previously with some modification.^30^ Briefly, female ZFDM *fa*/+ rats were superovulated by intraperitoneally injection with 15 IU (International Unit) of pregnant mare serum gonadotropin (NIPPON ZENYAKU KOGYO Co., Ltd.) and 15 IU of human chorionic gonadotropin (ASKA Pharmaceuticals Co., Ltd.), and then the female rats were mated with male ZFDM (*fa*/+ or *fa/fa*) rats. The next day, pronuclear-stage embryos were collected from superovulated rats and cultured in a modified Krebs-Ringer bicarbonate (m-KRB) culture medium before microinjection. The *Lcn2* target sequence (5′-TGACTACGACTAGTTTGCCA-3′; Fig. 3A) was designed using CRISPOR (http://crispor.gi.ucsc.edu). The recombinant Cas9 protein and crRNA and tracrRNA were purchased from Integrated DNA Technologies (Coralville, Iowa, USA). The chemically synthesized single-strand oligo-DNA (ssODN; 5′-AAGTGGCCGACACTGACTACGACCAGTTTGCCATGGTATTTTTCCAGAAGACCTCTGAAA-3′, Exigen) was used for replacing the nonsense mutation with the wild-type allele on the *Lcn2* locus. The recombinant Cas9 protein (50 ng/mL), chemically synthesized crRNA (25 ng/mL) and tracrRNA (25 ng/mL), and the ssODN (100 ng/μL) were co-injected into the cytoplasm of pronuclear stage embryos. The injected embryos were cultured in m-KRB culture medium overnight. The 2-cell embryos were transferred into the oviduct of pseudopregnant SD rats anaesthetized using a mixture of 0.15 mg/kg medetomidine, 2.0 mg/kg midazolam, and 2.5 mg/kg butorphanol during operation. After the surgery, 0.15 mg/kg atipamezole (NIPPON ZENYAKU KOGYO Co., Ltd.) and 5 mg/kg enrofloxacin (Kyoritsu Seiyaku) were administered. Analgesics (0.01 mg/kg buprenorphine; Otsuka Pharmaceutical Co. Ltd.) were administered subcutaneously twice on the day of surgery and the following day.

To confirm the genome-edited alleles, the Sanger sequencing analysis was performed on the target locus. The PCR products were obtained from tail tip genome DNAs by using the primer set (FW, 5′-aaccctgggtatgacctgaa-3′; RV, 5′-ctggggcctggattattgta-3′), and sequenced with each of the primers. Two female founder rats (G0-28 and -29) heterozygous for the genome-edited allele were obtained and were mated with male ZFDM rats to produce offsprings. The inheritance of the genome-edited allele was confirmed in the offsprings. Both lines were maintained for several generations to produce *fa/fa* animals homozygous or heterozygous for the genome-edited allele and those homozygous for the original mutant allele. Finally, the genome-edited allele was fixed in the homozygous state. The G0-28- and -29-derived genome-edited ZFDM rats, ZFDM-*Lcn2*^*em1Nyo*^, and -*Lcn2*^*em2Nyo*^, were deposited to the NBRP-Rat under deposition No.0972 and No.0973, respectively.

### Phenotyping

The rats were checked for body weights and nonfasting blood glucose levels by a portable glucose metre (ANTSENSE Duo, HORIBA, Ltd.). Diabetes was defined as blood glucose levels equal to or higher than 300 mg/dL for 3 consecutive weeks under ad libitum dietary conditions. The week of the diabetes onset was defined as the first week in which blood glucose levels were equal to or higher than 300 mg/dL.

### Genotyping of the *fatty* mutation in the leptin receptor gene

A PCR-RFLP system was used for genotyping the *fatty* mutation. PCR product (596 bp) amplified by using a primer set (FW, 5′-aagccatctcatttgctggt-3′; RV, 5′-ggcaggcagatctctcaatc-3′) is digested by restriction enzyme *Msp*I (CCGG). The 596 bp fragment (wildtype allele) is further divided into 328 bp and 268 bp fragments in the *fatty* allele.

### Plasma Lcn2 levels

Plasma Lcn2 levels were measured using ELISA kits (BioPorto Diagnostics A/S).

### Plasma lipid parameters

Plasma lipid parameters were measured using the medium-sized biochemistry automatic analyser 7180 (Hitachi, Ltd.).

### Genotyping of the missense variant in the growth hormone receptor gene

A PCR-RFLP system was developed for genotyping of the A546V missense variant found in ZFDM rats: PCR product (282 bp) amplified by using a primer set (FW, 5′-cagatgccaaaaagtgcatcgccg-3′; RV, 5′-ggtctgtgctcacatagccacat-3′) is digested by restriction enzyme *Acc*II (CGCG), resulting in 192 bp, 66 bp, and 24 bp fragments in ZF rats (wildtype allele) while 216 bp and 66 bp fragments in ZFDM rats (mutant allele).

### Genetic analysis

Female ZF *fa*/+ rats were crossed with male ZFDM *fa/fa* rats to produce F1 animals. Then, female ZFDM *fa*/+ rats were crossed with the male *fa/fa* F1 rats to produce backcross progenies. Male *fa/fa* backcross progenies were checked for nonfasting blood glucose levels until 60 weeks of age. Genotyping of the *Lcn2* Q137X nonsense mutation and the *Ghr* A546V missense variant were performed by the PCR-RFLP system as described above.

### Statistical analysis

Data are expressed as mean ± SEM (standard error of the mean). Differences among the groups were analysed with the Tukey–Kramer method as indicated in the figure legends. Association of the *Lcn2* genotype with diabetes was analysed with a chi-square test. *P* < 0.05 was regarded as statistically significant. Statistical analysis was performed using R (version 4.4.1).

## Results

### Comparative transcriptome analysis of the pancreatic islets between ZF and ZFDM rats

**Fig. 1.**
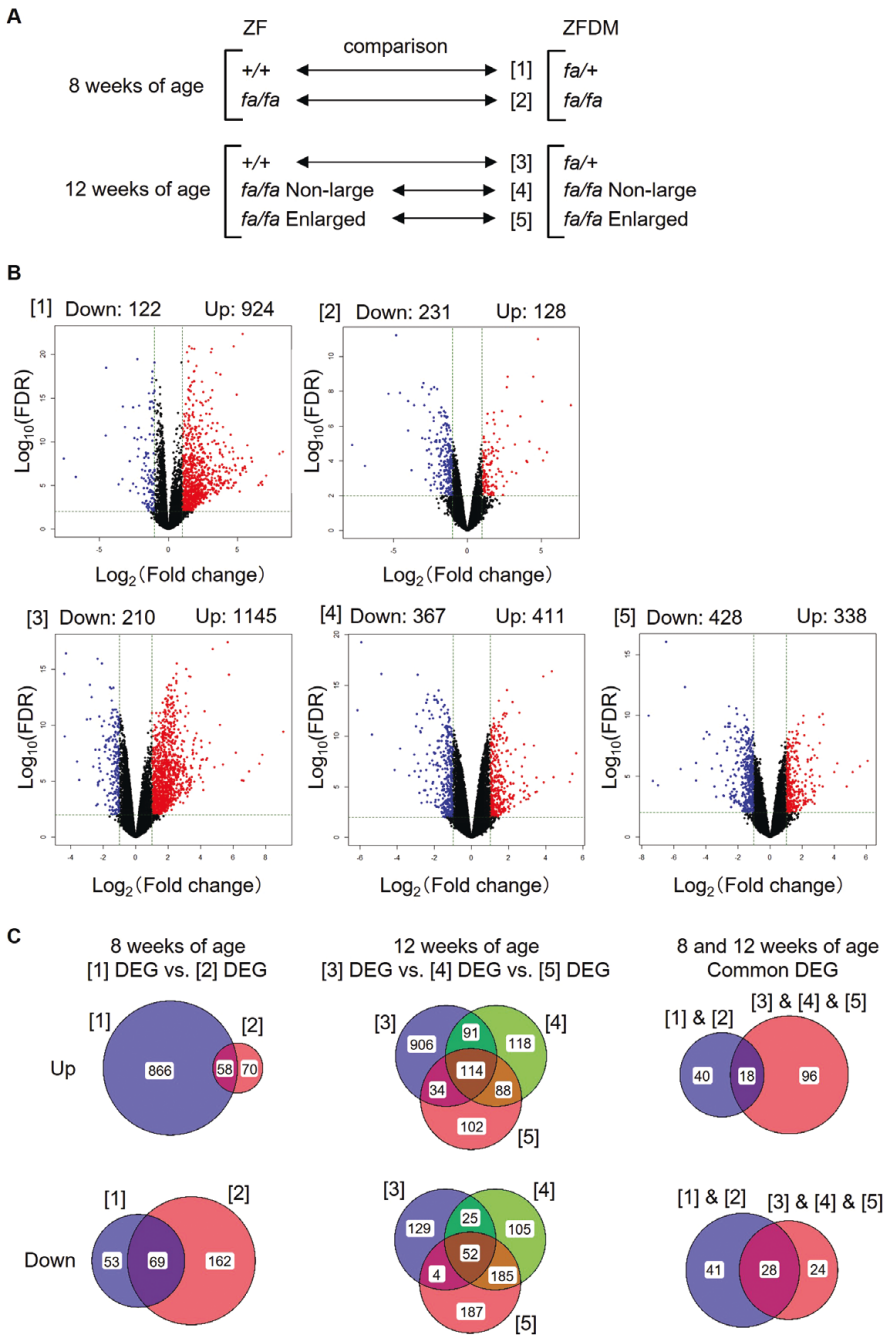
Comparative transcriptome analysis of the pancreatic islets between ZF and ZFDM rats. A) Comparisons of transcriptome ([1]–[5]) between male ZF and ZFDM rats at 8 and 12 weeks of age (*n* = 3 each). B) Volcano plots of the comparisons of transcriptome ([1]–[5]). Numbers of DEGs are shown as Down and Up in ZFDM rats as compared to ZF rats. C) Venn diagrams showing common DEGs.

To elucidate the gene expression profile of the pancreatic islets and primary genetic factors of T2D in ZFDM rats, we performed comparative transcriptome analysis between ZF and ZFDM rats at 8 and 12 weeks of age (Fig. 1A). ZFDM *fa/fa* rats at 8 weeks of age showed normoglycemia with very slight glucose intolerance upon oral glucose load, while those at 12 weeks of age exhibited apparent glucose intolerance.^6,7^ Since there was a large variation in the size of the islets in *fa/fa* rats at 12 weeks of age,^9^ we compared gene expression in non-large and enlarged (exceeding 300 μm in diameter) islets separately. Among ~13,000 genes detected, we found DEGs for each comparison (Fig. 1B): 1,046 DEGs for ZF +/+ versus ZFDM *fa/+* at 8 weeks of age (Supplementary Table S1), 359 DEGs for ZF *fa/fa* versus ZFDM *fa/fa* at 8 weeks of age (Supplementary Table S2), 1,355 DEGs for ZF +/+ versus ZFDM *fa/+* at 12 weeks of age (Supplementary Table S3), 778 DEGs for non-large islets of ZF *fa/fa* versus ZFDM *fa/fa* at 12 weeks of age (Supplementary Table S4), 766 DEGs for enlarged islets of ZF *fa/fa* versus ZFDM *fa/fa* at 12 weeks of age (Supplementary Table S5). Common DEGs at each age may represent primary expression differences between strains irrespective of obesity and subsequent glucose intolerance conditions, which may be reflected by primary genetic differences. We performed gene enrichment analysis on these common DEGs. Common 127 DEGs (up: 58, down: 69) at 8 weeks of age (Fig. 1C; Supplementary Table S6) were enriched in the GO term associated with ‘response to stimulus’ and ‘extracellular matrix’ (Supplementary Table S7). Common 166 DEGs (up: 114, down: 52) at 12 weeks of age (Fig. 1C; Supplementary Table S8) were enriched in the GO term associated with ‘positive regulation of multicellular organismal process’, ‘response to stimulus’, and ‘extracellular matrix’ (Supplementary Table S9). Gene enrichment analyses using the DAVID and Metascape tools produced similar results. Due to the relatively small number of genes, there was no significant GO term for common 46 DEGs (up: 18, down: 28) for all the comparisons (Fig. 1C; Supplementary Tables S10 and S11). However, these 46 DEGs may serve as strong candidate genes for T2D in ZFDM rats.

### Identification of a nonsense mutation in the *Lcn2* gene in the ZFDM rat

**Fig. 2.**
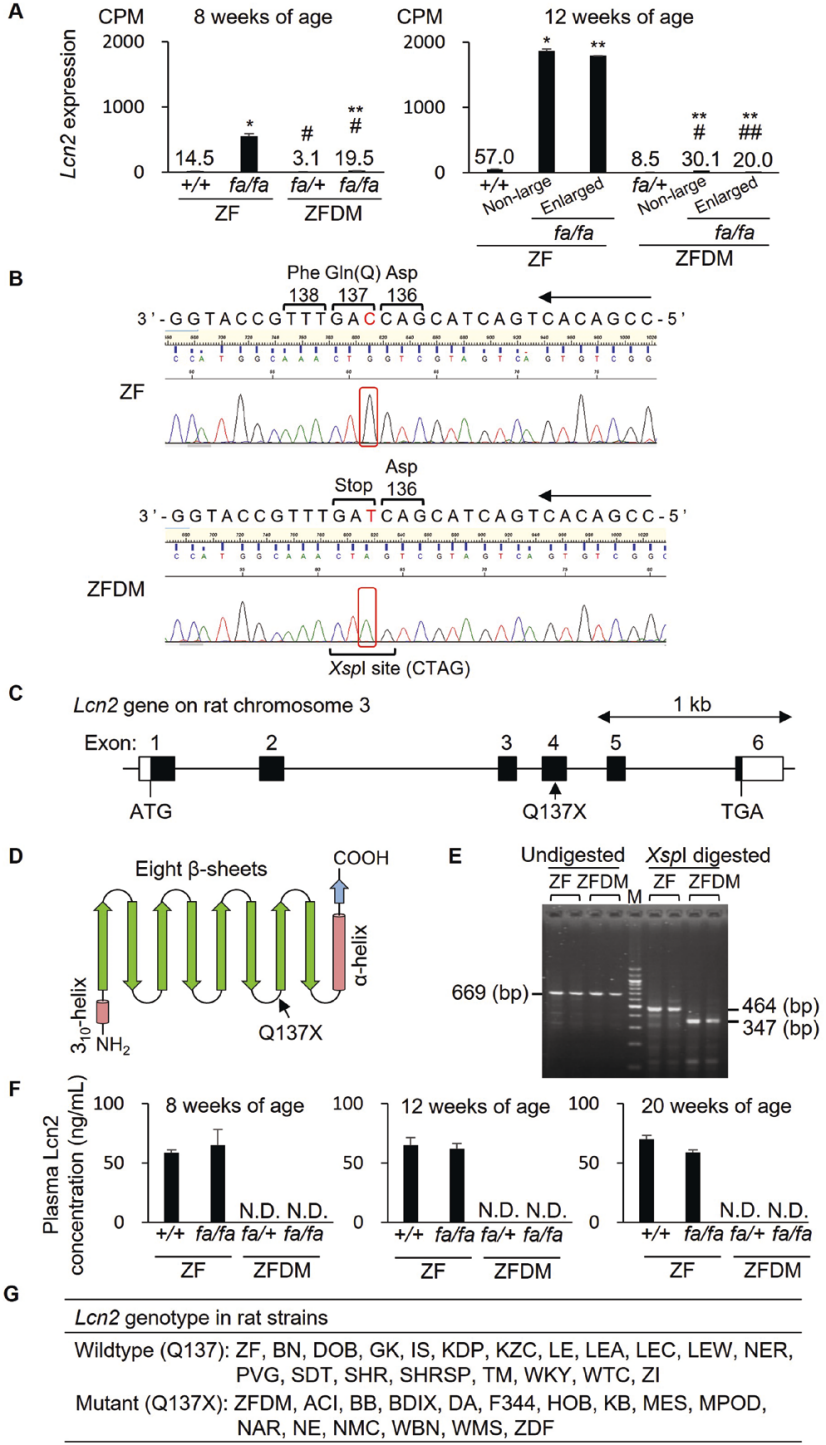
Characterization of the Q137X nonsense mutation in the *Lcn2* gene in ZFDM rats. A) Expression levels of *Lcn* in male ZF and ZFDM rats at 8 and 12 weeks of age. The data are CPM values derived from the RNA-sequencing analysis and expressed as means ± SEM (*n* = 3 each). Tukey-Kramer method was used for evaluation of statistical significance: *P < 0.05, **P < 0.01 (vs. +/+ or *fa/+* of each strain); #*P* < 0.05, ##*P* < 0.01 (vs. the corresponding group of ZF rats). B) Electropherogram of the sequencing data containing the Q137X nonsense mutation in the *Lcn2* gene. The mutation produces an *X*spI restriction site. C) Schematic diagram of the exon-intron structure of *Lcn2* gene in rat chromosome 3. D) Schematic diagram of the secondary structure of Lcn2 protein. E) Electrophoretic gel image of the genotyping by PCR-RFLP analysis. *Xsp*I digestion of the 669 bp PCR fragment produces 464 bp and 347 bp fragments in ZF and ZFDM rats, respectively. M, 100 bp DNA ladder marker. F) Plasma Lcn2 levels in male ZF and ZFDM rats at 8, 12, and 20 weeks of age. The data are expressed as means ± SEM (*n* = 3 each). Lcn2 protein was not detected (N.D.) in ZFDM rats. G) *Lcn2* genotypes in rat strains. See S13 Table for details.

To further elucidate primary genetic factors of T2D in ZFDM rats, we performed mutation analysis on RNA-seq data of the islets of ZF and ZFDM rats at 8 weeks of age and found that there were ~5,900 variants including 5 stop-gained variants (3 genes) and 921 missense variants as compared with the rat reference genome sequence (Supplementary Table S12). The ZF rat has stop-gained variants in *Commd7* and *RT1-Db1* while the ZFDM rat has those in *Lcn2* and *RT1-Db1*. Among genes with stop-gained variants, lipocalin 2 (*Lcn2*) was included in the common 46 DEGs. In addition, the ZFDM rat has 2 missense variants (K541N and A546V) in the growth hormone receptor (*Ghr*) gene which was also included in the common 46 DEGs. Since stop-gained variants would have a more significant effect on the gene function as compared with missense variants, we focussed on *Lcn2*. In ZF rats, *Lcn2* gene expression was much higher in the islets of *fa/fa* rats as compared with that of +/+ rats, and the expression levels increased with age (Fig. 2A). In contrast, the expression was significantly lower in the islets of both *fa*/+ and *fa/fa* in the ZFDM rat as compared with those of ZF rats. As for ZF rats, the expression levels in ZFDM *fa/fa* rats were higher than those in ZFDM *fa*/+ rats, but the difference was much lower than that of ZF rats.

Sanger sequencing confirmed the stop-gained variant in *Lcn2* and verified that the ZFDM rat has a nonsense mutation at the 137th glutamine codon (CAG to TAG), c.409C > T; p.Q137X (Fig. 2B). *Lcn2* consists of 6 exons spanning ~3.3 kb genomic region on rat chromosome 3 and the Q137X mutation is located on exon 4 (Fig. 2C). Lcn2 protein belongs to the lipocalin family, and the protein structure is characterized by a single polypeptide chain that forms a barrel-like structure composed of 8 β-sheets, which create a central cavity (Fig. 2D). The mutation disrupts the protein after the sixth β-sheets, deleting the seventh and eighth β-sheets and C-terminal α-helix domain. The mutant and wildtype alleles were clearly distinguished by PCR-RFLP analysis (Fig. 2E). Since Lcn2 is known to be secreted into the blood, plasma Lcn2 levels were examined in ZF and ZFDM rats. Plasma Lcn2 proteins were detected in both +/+ and *fa/fa* of the ZF rat with no significant changes with age. In contrast, Lcn2 proteins were hardly detected in the plasma of both *fa*/+ and *fa/fa* of the ZFDM rat (Fig. 2F).

To clarify the frequency of the mutation in rat species, we searched for the mutation among 159 inbred and 2 outbred rat strains. Interestingly, the mutant allele was detected in 32 inbred and 1 outbred strains including well-known inbred rat strains such as BB, DA, and F344 (Fig. 2G; Supplementary Table S13).

### Evaluation of the relevance of the *Lcn2* mutation with T2D by genome editing technique

**Fig. 3.**
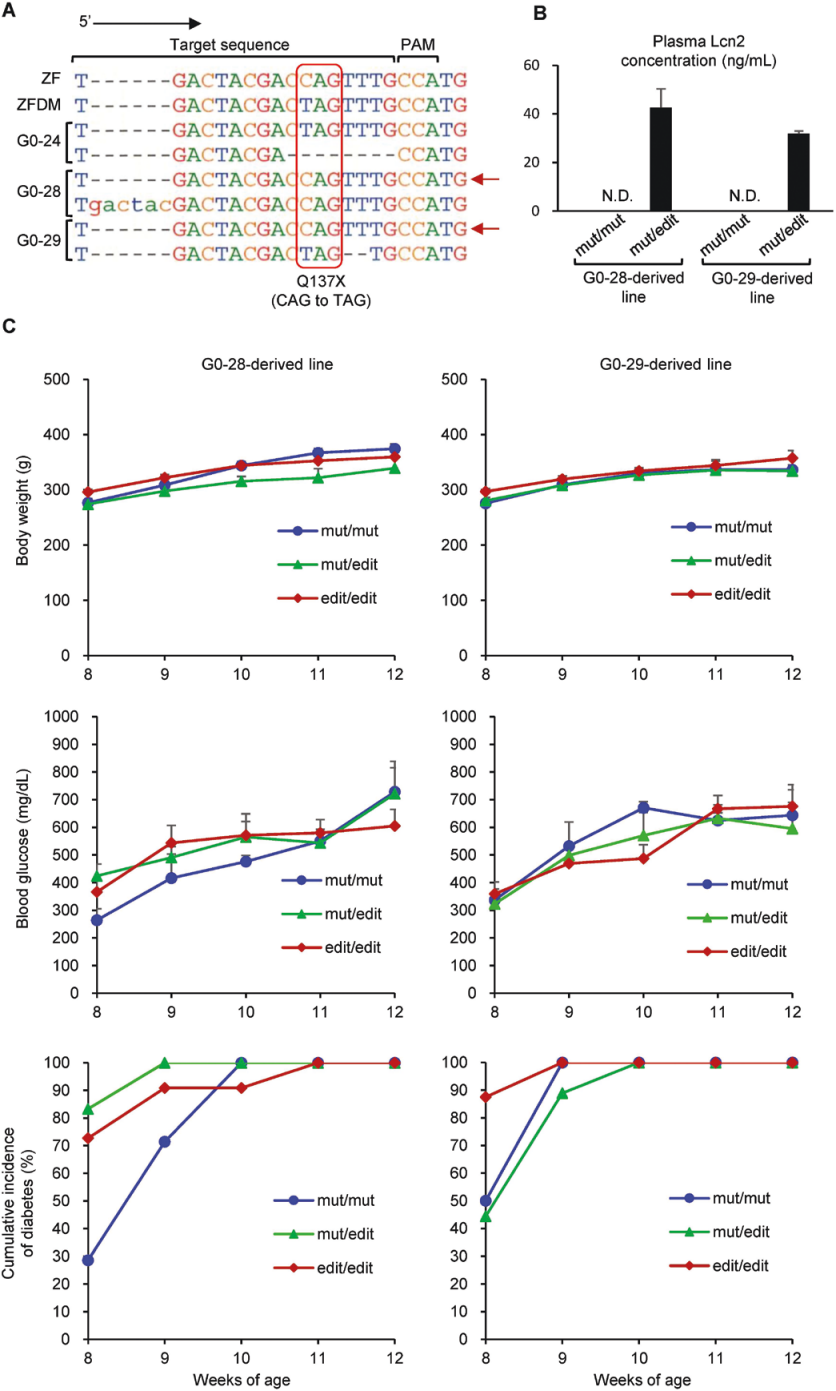
Generation and characterization of *Lcn2* knock-in ZFDM rats. A) Sequence alignment for the target sequence of genome editing. Arrows indicate the correctly edited alleles. B) Plasma Lcn2 levels in male progenies in G0-28- and -29-derived lines: mut, the mutant allele; edit, the correctly edited allele. The data are expressed as means ± SEM (*n* = 5 each, except for mut/edit in G0-29-derived line: *n* = 4). Lcn2 protein was not detected (N.D.) in animals homozygous for the mutant allele. C) Body weights, blood glucose levels, and cumulative incidence of diabetes in male *fa/fa* progenies in G0-28- and -29-derived lines: mut, the mutant allele; edit, the correctly edited allele. The data are expressed as means ± SEM (*n* = 6–12).

To evaluate directly that the *Lcn2* mutation is responsible for the development of T2D in ZFDM rats, we generated *Lcn2* knock-in rats to correct the stop-gained variant in the *Lcn2* gene in ZFDM rats using CRISPR/Cas9-mediated genome editing. Among ~30 founder (G0) animals, there were 2 animals (G0-28 and -29) harbouring an edited allele in which the nonsense mutation was replaced with a wild-type nucleotide (Fig. 3A). Successful correction of the Q137X mutation was confirmed by the fact that animals in both G0-28- and -29-derived lines heterozygous for the edited allele showed the expression of the Lcn2 protein in the plasma (Fig. 3B). We then produced male *fa/fa* animals homozygous or heterozygous for the edited allele and compared phenotypes with animals homozygous for the mutant allele (Fig. 3C). There was no significant difference in body weights among animals with the 3 genotypes. Blood glucose levels also showed no significant difference among the 3 genotypes. Accordingly, there was no significant difference in the cumulative incidence of diabetes among them. Although Lcn2 has been reported to be related with plasma lipid levels in humans,^31^ there was no clear difference in plasma lipid parameters among these animals (Supplementary Fig. S1).

### Genetic linkage analysis of the *Lcn2* mutation with T2D

**Fig. 4.**
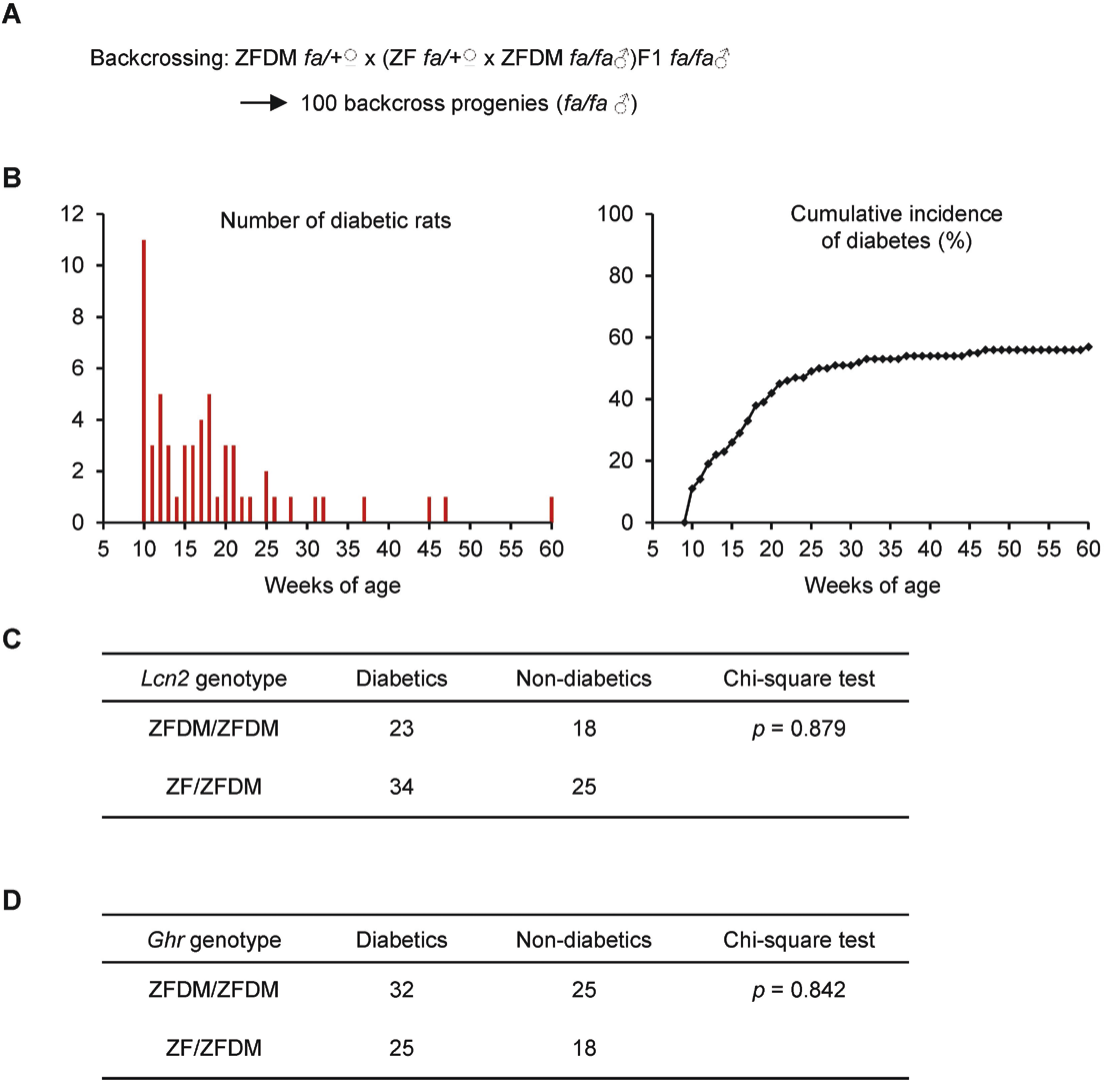
Genetic linkage analysis of the *Lcn2* mutation and the *Ghr* missense variant with the onset of T2D. A) Overview of the method producing backcross progenies between ZF and ZFDM rats. B) Number of diabetic rats (left) and cumulative incidence of diabetes (right) in the backcross progenies till 60 weeks of age. C and D) Chi-square test for the association of the *Lcn2* genotype (C) or the *Ghr* genotype (D) with diabetic phenotype in the backcross progenies.

To further confirm the relevance of the *Lcn2* mutation with T2D, we also performed a genetic linkage analysis by using a genetic cross between ZF and ZFDM rats. Since none of the male F1 animals homozygous for *fa/fa* developed diabetes, we produced backcross progenies by crossing between female ZFDM and male F1 (Fig. 4A). Among 100 male backcross progenies (*fa/fa*), 57 animals developed diabetes by 60 weeks of age, suggesting that a recessively acting autosomal allele in ZFDM rats is involved in the development of T2D in the cross (Fig. 4B). We determined the *Lcn2* genotypes in each backcross progeny and performed a chi-square test between the genotype and diabetic phenotype (Fig. 4C). It has been revealed that there was no significant relationship of the *Lcn2* genotype with the onset of diabetes. In addition, we also examined the relevance of the *Ghr* missense variants with T2D and clarified that there was also no significant relationship with T2D (Fig. 4D).

## Discussion

In the present study, by using ZFDM rat as a model of obese T2D, we found that (i) 46 genes exhibit significant expression differences between ZF and ZFDM islets irrespective of obesity and glucose intolerance states; (ii) among these common DEGs, the ZFDM rat has a nonsense mutation in the *Lcn2* gene, the mutation is distributed widely in rat species; and (iii) the *Lcn2* mutation is, however, not involved in the development of T2D.

At first, we performed a comparative transcriptome analysis of the pancreatic islets between ZF and ZFDM rats at 8 and 12 weeks of age. The common DEGs of the comparison (ZF +/+ vs. ZFDM *fa*/+) and (ZF *fa/fa* vs. ZFDM *fa/fa*) at each age group were associated with ‘extracellular matrix’: *Fgb, Fgg, Hapln4, Lgals3, Mmp13, Mmp19,* and *P3h2* showed lower expression, while *Adamts16, Ccn4, Col7a1, Col9a1, Cspg4, Cthrc1, Fmod, Olfml2a, Ptx3, S100a4, S100a6, Serpinf1, Spon2, Srpx2, Tgfb1, Tnn,* and *Wnt5a* showed higher expression in ZFDM rats as compared with ZF rats.

Matrix metalloproteinases (MMPs) degrade collagenous extracellular matrix, which are associated with tissue remodelling and fibrosis. In ZDF rats, as compared with lean control rats, gene expressions of *Mmp2*, *-12*, and *-14* in diabetic *fa/fa* rats were increased with the onset of islet dysfunction and diabetes.^4^ In ZFDM rats, we found that most of *Mmps* exhibit higher expressions in *fa/fa* rats as compared with +/+ or *fa/+* rats (Supplementary Fig. S2A). In addition, the expressions of most *Mmps* are higher in ZFDM rats as compared with those in ZF rats, while the expressions of *Mmp13* and *Mmp19* show opposite profiles (Supplementary Fig. S2B), indicating that these *Mmps* might have some roles in suppressing the development of islet dysfunction and diabetes.

Gene expression profiles of collagens (Supplementary Fig. S3A), the main component of extracellular matrix, show a similar pattern with those of *Mmps*, indicating that *fa/fa* animals have more extracellular matrix than +/+ or *fa/+* animals, the degree is higher in ZFDM rats as compared with that in ZF rats. The expressions of *Col7a1* and *Col9a1* are significantly higher in ZFDM rats as compared with those in ZF rats (Supplementary Fig. S3B). Since these collagens consist of minor collagen components, the roles in islet dysfunction and diabetes remain to be elucidated. However, the common DEGs for all the comparisons may correspond to primary gene expression differences between strains irrespective of obesity and subsequent glucose intolerance conditions, which could represent primary genetic differences.

Secondly, we therefore searched for functional variants in genes expressed in ZF and ZFDM islets. Among a total of 5 stop-gained variants, only *Lcn2* was found to be included in the common 46 DEGs and ZFDM has a Q137X nonsense mutation, serving *Lcn2* as a strong candidate for T2D. The mutation deletes significant C-terminal domains of the protein, which may lead to a loss of function of the protein. In addition, the gene expression of the mutant *Lcn2* was strongly reduced due to the nonsense-mediated mRNA decay, resulting in no detectable protein in the plasma. These findings indicate that the Q137X nonsense mutation causes a deficiency of the Lcn2 protein.

To our surprise, the *Lcn2* mutation is revealed to be distributed widely in inbred rat strains including BB, a model of type 1 diabetes (T1D); DA, a model of collagen-induced arthritis, adjuvant-induced arthritis, and experimental autoimmune encephalomyelitis; F344, a well-used control strain; NAR, a model of analbuminemia; WBN, a model of nonobese T2D; and ZDF, a model of obese T2D. Especially, the fact that F344 strains have the mutation needs to be noticed since F344 strains frequently serve as background strains for genome editing experiments and as standard strains for preclinical drug safety assessment. Regarding models of diabetes, T1D model KDP rat and nonobese T2D model SDT rat have wildtype alleles, suggesting no apparent association of the mutation with both T1D and T2D. By database search, we found that the mutation has already been registered as rs3323635808 in public databases such as the Rat Genome Database (https://rgd.mcw.edu/rgdweb/homepage/), along with information on its widespread distribution in 50 laboratory rat strains including ACI, DA, F344, M520, and WN.

Finally, we evaluated the relevance of the *Lcn2* mutation with T2D by both genome editing and genetic linkage analysis. Our analyses revealed that the mutation is not involved in the development of T2D in ZFDM rats. In addition to adipose tissue, liver, and immune cells that primarily produce Lcn2, various tissues and cells, including pancreatic β-cells, also produce Lcn2 under inflammation or stress conditions.^32^ Lcn2 is thought to have a beneficial role in the regulation of various aspects of energy metabolism: protection from diet-induced obesity and insulin resistance,^33^ high-fat diet-induced adipose tissue remodelling,^34^ and brown fat activation.^35^ Serum LCN2 levels positively correlate with energy expenditure and fatty acid oxidation in normal weight but not obese women.^36^ In contrast, other reports showed that serum Lcn2 levels are associated with obesity and insulin resistance in humans and mice.^37–39^

Lcn2 has been also reported as a bone-derived hormone with metabolic regulatory effects^40^: osteoblast-derived Lcn2 maintains glucose homeostasis by inducing insulin secretion, improving glucose tolerance and insulin sensitivity, and inhibiting food intake. In addition, Lcn2 counteracts metabolic dysregulation in obesity and diabetes, suggesting a distinct beneficial effect of Lcn2 on β-cell function and adaptive β-cell proliferation during toxicity or onset of obesity.^41^ A model of the compensatory homeostatic role of Lcn2 has been proposed, in which Lcn2 counteracts insulin resistance progression, prevents obesity, and suppresses diabetes, while once this mechanism is overwhelmed, obesity increases and diabetes develops.^41^ Taken together, our findings in the ZFDM rat, an extreme obese and severe T2D model, suggest that the compensatory homeostatic mechanism is overwhelmed and Lcn2, therefore, could not exert any beneficial effects on β-cell function and proliferation during the development of obesity and T2D. The role of Lcn2 needs to be examined in other rat models of mild obesity or diabetes.

In conclusion, we here find a nonsense mutation in the *Lcn2* gene in a rat model of obese T2D, the mutation is distributed widely in rat species. Although we could not show the relevance of the *Lcn2* mutation with the development of T2D, several rat strains would serve as *Lcn2* deficient models, contributing to unravel normal and pathophysiological roles of Lcn2 in a wide variety of phenotypes.

## Supplementary Material

dsaf004_suppl_Supplementary_Figures

dsaf004_suppl_Supplementary_Table_S1

dsaf004_suppl_Supplementary_Table_S2

dsaf004_suppl_Supplementary_Table_S3

dsaf004_suppl_Supplementary_Table_S4

dsaf004_suppl_Supplementary_Table_S5

dsaf004_suppl_Supplementary_Table_S6

dsaf004_suppl_Supplementary_Table_S7

dsaf004_suppl_Supplementary_Table_S8

dsaf004_suppl_Supplementary_Table_S9

dsaf004_suppl_Supplementary_Table_S10

dsaf004_suppl_Supplementary_Table_S11

dsaf004_suppl_Supplementary_Table_S12

dsaf004_suppl_Supplementary_Table_S13

## Data Availability

All raw RNA sequencing data have been deposited in the DDBJ Sequence Read Archive (DRA; https://www.ddbj.nig.ac.jp/dra/index-e.html) under the accession numbers PRJDB7245 and PRJDB12053.

## References

[1] Bacos K , et al Type 2 diabetes candidate genes, including PAX5, cause impaired insulin secretion in human pancreatic islets. J Clin Invest. 2023:133:e163612. https://doi.org/10.1172/JCI163612 36656641 PMC9927941

[2] Neelankal John A , Ram R, Jiang FX. RNA-Seq analysis of islets to characterise the dedifferentiation in type 2 diabetes model mice db/db. Endocr Pathol. 2018:29:207–221. https://doi.org/10.1007/s12022-018-9523-x 29542001

[3] Griffen SC , Wang J, German MS. A genetic defect in beta-cell gene expression segregates independently from the fa locus in the ZDF rat. Diabetes. 2001:50:63–68. https://doi.org/10.2337/diabetes.50.1.63 11147796

[4] Zhou YP , et al Matrix metalloproteinases contribute to insulin insufficiency in Zucker diabetic fatty rats. Diabetes. 2005:54:2612–2619. https://doi.org/10.2337/diabetes.54.9.2612 16123349

[5] Li X , et al Islet microvasculature in islet hyperplasia and failure in a model of type 2 diabetes. Diabetes. 2006:55:2965–2973. https://doi.org/10.2337/db06-0733 17065332

[6] Yokoi N , et al A novel rat model of type 2 diabetes: the Zucker fatty diabetes mellitus ZFDM rat. J Diabetes Res. 2013:2013:103731. https://doi.org/10.1155/2013/103731 23671847 PMC3647587

[7] Gheni G , et al Characterization of the prediabetic state in a novel rat model of type 2 diabetes, the ZFDM Rat. J Diabetes Res. 2015:2015:261418. https://doi.org/10.1155/2015/261418 25961052 PMC4415487

[8] Nakanishi S , Kuramoto T, Kashiwazaki N, Yokoi N. Genetic profiling of two phenotypically distinct outbred rats derived from a colony of the Zucker fatty rats maintained at Tokyo Medical University. Exp Anim. 2017:66:91–98. https://doi.org/10.1538/expanim.16-0068 27795491 PMC5411295

[9] Hayami T , et al Tumor-like features of gene expression and metabolic profiles in enlarged pancreatic islets are associated with impaired incretin-induced insulin secretion in obese diabetes: a study of Zucker fatty diabetes mellitus rat. J Diabetes Investig. 2020:11:1434–1447. https://doi.org/10.1111/jdi.13272 PMC7610108 32279428

[10] Okeda T , Ono J, Takaki R, Todo S. Simple method for the collection of pancreatic islets by the use of Ficoll-Conray gradient. Endocrinol Jpn. 1979:26:495–499. https://doi.org/10.1507/endocrj1954.26.495 387387

[11] Carter JD , et al A practical guide to rodent islet isolation and assessment. Biol Proced Online. 2009:11:3–31. https://doi.org/10.1007/s12575-009-9021-0 19957062 PMC3056052

[12] Bolger AM , Lohse M, Usadel B. Trimmomatic: a flexible trimmer for Illumina sequence data. Bioinformatics. 2014:30:2114–2120. https://doi.org/10.1093/bioinformatics/btu170 24695404 PMC4103590

[13] Andrews, S. 2010. FastQC: a quality control tool for highthroughput sequence data. http://www.bioinformatics.babraham.ac.uk/projects/fastqc/

[14] Dobin A , et al STAR: ultrafast universal RNA-seq aligner. Bioinformatics. 2013:29:15–21. https://doi.org/10.1093/bioinformatics/bts635 23104886 PMC3530905

[15] Danecek P , et al Twelve years of SAMtools and BCFtools. GigaScience. 2021:10:giab008. https://doi.org/10.1093/gigascience/giab008 33590861 PMC7931819

[16] Liao Y , Smyth GK, Shi W. featureCounts: an efficient general purpose program for assigning sequence reads to genomic features. Bioinformatics. 2014:30:923–930. https://doi.org/10.1093/bioinformatics/btt656 24227677

[17] Robinson MD , McCarthy DJ, Smyth GK. edgeR: a Bioconductor package for differential expression analysis of digital gene expression data. Bioinformatics. 2010:26:139–140. https://doi.org/10.1093/bioinformatics/btp616 19910308 PMC2796818

[18] Chen Y , Lun AT, Smyth GK. From reads to genes to pathways: differential expression analysis of RNA-Seq experiments using Rsubread and the edgeR quasi-likelihood pipeline. F1000Res. 2016:5:1438. https://doi.org/10.12688/f1000research.8987.2 27508061 PMC4934518

[19] Durinck S , et al BioMart and Bioconductor: a powerful link between biological databases and microarray data analysis. Bioinformatics. 2005:21:3439–3440. https://doi.org/10.1093/bioinformatics/bti525 16082012

[20] Durinck S , Spellman PT, Birney E, Huber W. Mapping identifiers for the integration of genomic datasets with the R/Bioconductor package biomaRt. Nat Protoc. 2009:4:1184–1191. https://doi.org/10.1038/nprot.2009.97 19617889 PMC3159387

[21] Huang da W , Sherman BT, Lempicki RA. Systematic and integrative analysis of large gene lists using DAVID bioinformatics resources. Nat Protoc. 2009:4:44–57. 19131956 10.1038/nprot.2008.211

[22] Sherman BT , et al DAVID: a web server for functional enrichment analysis and functional annotation of gene lists (2021 update). Nucleic Acids Res. 2022:50:W216–W221. https://doi.org/10.1093/nar/gkac194 35325185 PMC9252805

[23] Zhou Y , et al Metascape provides a biologist-oriented resource for the analysis of systems-level datasets. Nat Commun. 2019:10:1523. https://doi.org/10.1038/s41467-019-09234-6 30944313 PMC6447622

[24] McKenna A , et al The Genome Analysis Toolkit: a MapReduce framework for analyzing next-generation DNA sequencing data. Genome Res. 2010:20:1297–1303. https://doi.org/10.1101/gr.107524.110 20644199 PMC2928508

[25] DePristo MA , et al A framework for variation discovery and genotyping using next-generation DNA sequencing data. Nat Genet. 2011:43:491–498. https://doi.org/10.1038/ng.806 21478889 PMC3083463

[26] Van der Auwera GA , O’Connor BD. Genomics in the Cloud: Using Docker, GATK, and WDL in Terra. 1st ed. O’Reilly Media, Inc; 2020.

[27] Danecek P , et al; 1000 Genomes Project Analysis Group. The variant call format and VCFtools. Bioinformatics. 2011:27:2156–2158. https://doi.org/10.1093/bioinformatics/btr330 21653522 PMC3137218

[28] McLaren W , et al The Ensembl variant effect predictor. Genome Biol. 2016:17:122. https://doi.org/10.1186/s13059-016-0974-4 27268795 PMC4893825

[29] Fuse M , et al Identification of a major locus for islet inflammation and fibrosis in the spontaneously diabetic Torii rat. Physiol Genomics. 2008:35:96–105. https://doi.org/10.1152/physiolgenomics.90214.2008 18612083

[30] Yoshimi K , et al ssODN-mediated knock-in with CRISPR-Cas for large genomic regions in zygotes. Nat Commun. 2016:7:10431. https://doi.org/10.1038/ncomms10431 26786405 PMC4736110

[31] Wallenius V , et al The lipocalins retinol-binding protein-4, lipocalin-2 and lipocalin-type prostaglandin D2-synthase correlate with markers of inflammatory activity, alcohol intake and blood lipids, but not with insulin sensitivity in metabolically healthy 58-year-old Swedish men. Exp Clin Endocrinol Diabetes. 2011:119:75–80. https://doi.org/10.1055/s-0030-1265212 21104585

[32] Kim J , Oh CM, Kim H. The interplay of adipokines and pancreatic beta cells in metabolic regulation and diabetes. Biomedicines. 2023:11:2589. https://doi.org/10.3390/biomedicines11092589 37761031 PMC10526203

[33] Guo H , et al Lipocalin-2 deficiency impairs thermogenesis and potentiates diet-induced insulin resistance in mice. Diabetes. 2010:59:1376–1385. https://doi.org/10.2337/db09-1735 20332347 PMC2874698

[34] Guo H , et al Evidence for the regulatory role of lipocalin 2 in high-fat diet-induced adipose tissue remodeling in male mice. Endocrinology. 2013:154:3525–3538. https://doi.org/10.1210/en.2013-1289 23885013 PMC3776868

[35] Zhang Y , et al Lipocalin 2 regulates brown fat activation via a nonadrenergic activation mechanism. J Biol Chem. 2014:289:22063–22077. https://doi.org/10.1074/jbc.M114.559104 24917675 PMC4139221

[36] Paton CM , et al Lipocalin-2 increases fat oxidation in vitro and is correlated with energy expenditure in normal weight but not obese women. Obesity (Silver Spring). 2013:21:E640–E648. https://doi.org/10.1002/oby.20507 23640923

[37] Yan QW , et al The adipokine lipocalin 2 is regulated by obesity and promotes insulin resistance. Diabetes. 2007:56:2533–2540. https://doi.org/10.2337/db07-0007 17639021

[38] Wang Y , et al Lipocalin-2 is an inflammatory marker closely associated with obesity, insulin resistance, and hyperglycemia in humans. Clin Chem. 2007:53:34–41. https://doi.org/10.1373/clinchem.2006.075614 17040956

[39] Rashad NM , El-Shal AS, Etewa RL, Wadea FM. Lipocalin-2 expression and serum levels as early predictors of type 2 diabetes mellitus in obese women. IUBMB Life. 2017:69:88–97. https://doi.org/10.1002/iub.1594 28116808

[40] Mosialou I , et al MC4R-dependent suppression of appetite by bone-derived lipocalin 2. Nature. 2017:543:385–390. https://doi.org/10.1038/nature21697 28273060 PMC5975642

[41] Mosialou I , et al Lipocalin-2 counteracts metabolic dysregulation in obesity and diabetes. J Exp Med. 2020:217:e20191261. https://doi.org/10.1084/jem.20191261 32639539 PMC7537391

