# Supplementary material for: Comparative transcriptome and mutation analyses of the pancreatic islets of a rat model of obese type 2 diabetes identifies a frequently distributed nonsense mutation in the lipocalin 2 gene
Source: DNA Res. 2025 Feb 26;32(2):dsaf004. doi: 10.1093/dnares/dsaf004 (PMC11976058; doi:10.1093/dnares/dsaf004)
Supplement: dsaf004_suppl_Supplementary_Figures [file dsaf004_suppl_supplementary_figures.pdf]

Description of supplementary figures

Supplementary Figure S1. Plasma lipid parameters in G0-28- and -29-derived lines.

Supplementary Figure S2. Gene expression profiles of matrix metalloproteinases (MMPs) in ZF and ZFDM rats at 12 weeks of age.

Supplementary Figure S3. Gene expression profiles of collagens in ZF and ZFDM rats at 12 weeks of age.

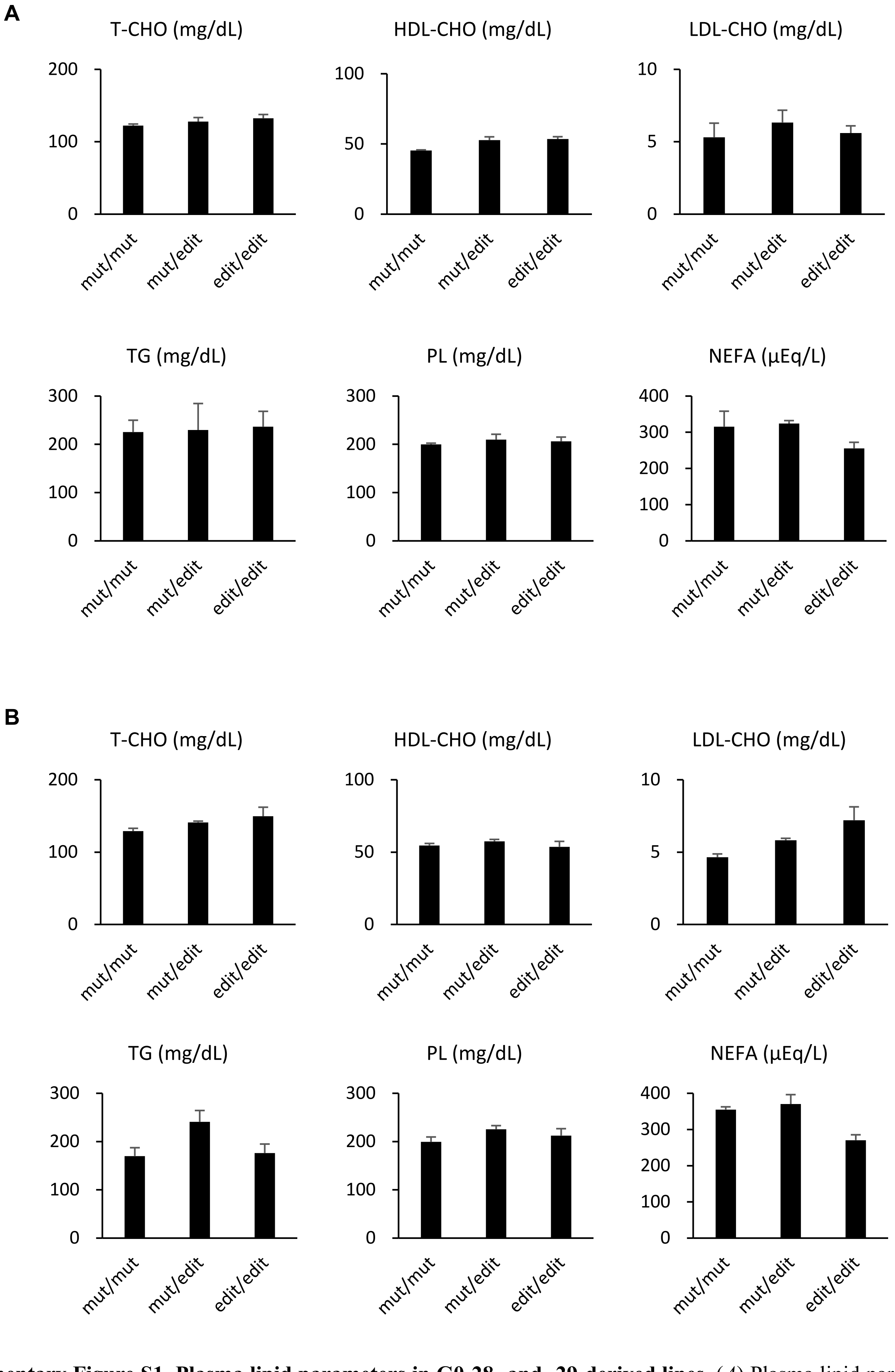

Supplementary Figure S1. Plasma lipid parameters in G0-28- and -29-derived lines. (*A*) Plasma lipid parameters in G0-28-derived lines. (*B*) Plasma lipid parameters in G0-29-derived lines. Data are expressed as means ± SEM (n=4-5 each). mut, the mutant allele; edit, the correctly edited allele. T-CHO, total cholesterol; HDL-CHO, HDL cholesterol; LDL-CHO, LDL cholesterol; TG, triglyceride; PL, phospholipid; NEFA, non esterified fatty acid.

A

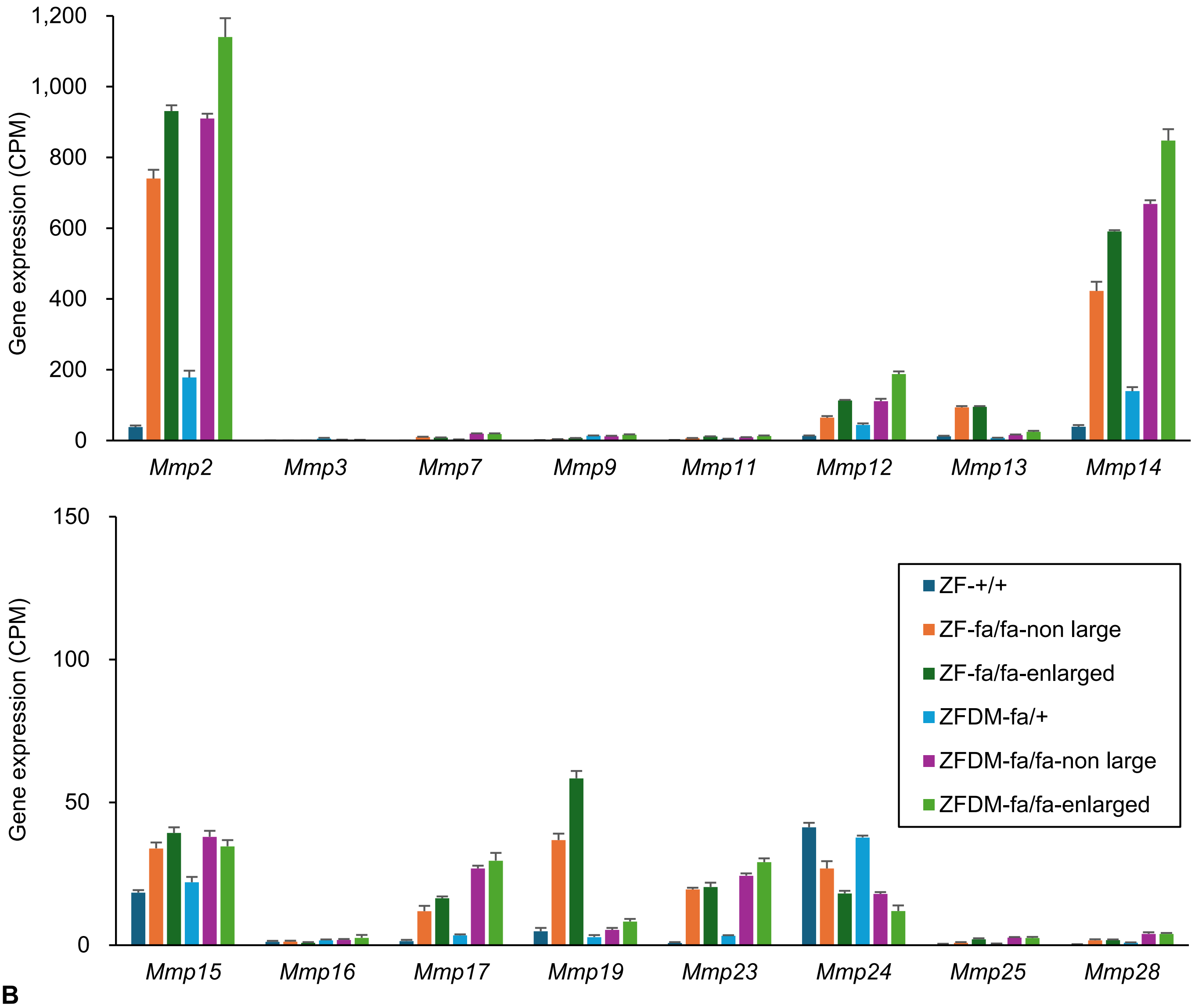

B

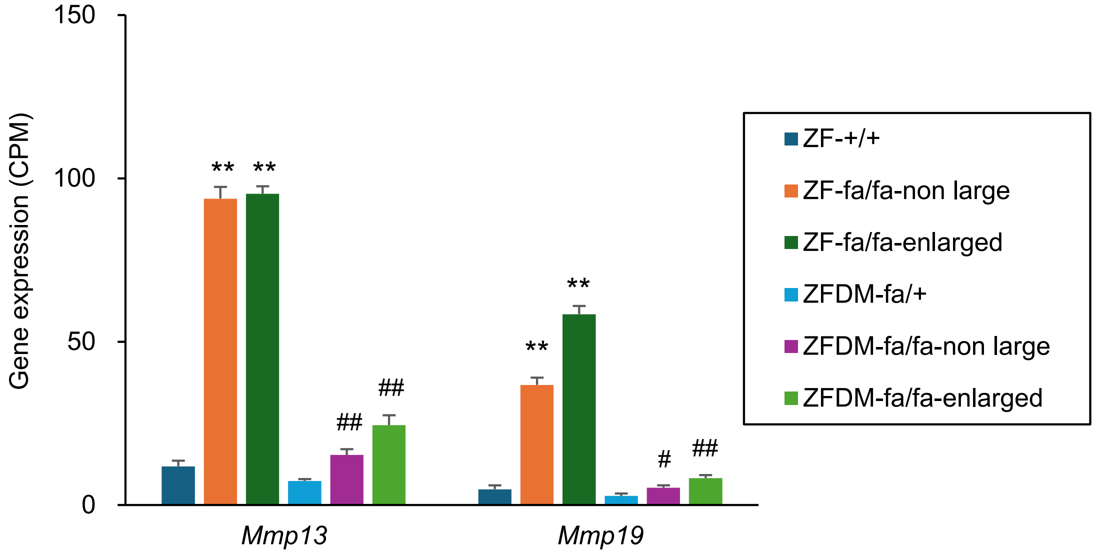

Supplementary Figure S2. Gene expression profiles of matrix metalloproteinases (MMPs) in ZF and ZFDM rats at 12 weeks of age. (*A*) Expression levels of *Mmps* in ZF and ZFDM rats at 12 weeks of age. The data are CPM values derived from the RNA-sequencing analysis and expressed as means ± SEM (n=3 each). (*B*) Expression levels of *Mmp13* and *Mmp19* in ZF and ZFDM rats at 12 weeks of age. The data are CPM values derived from the RNA-sequencing analysis and expressed as means ± SEM (n=3 each). Tukey-Kramer method was used for evaluation of statistical significance: ***P* < 0.01 (vs. +/+ or *fa*/+ of each strain); #*P* < 0.05, ##*P* < 0.01 (vs. the corresponding group of ZF rats).

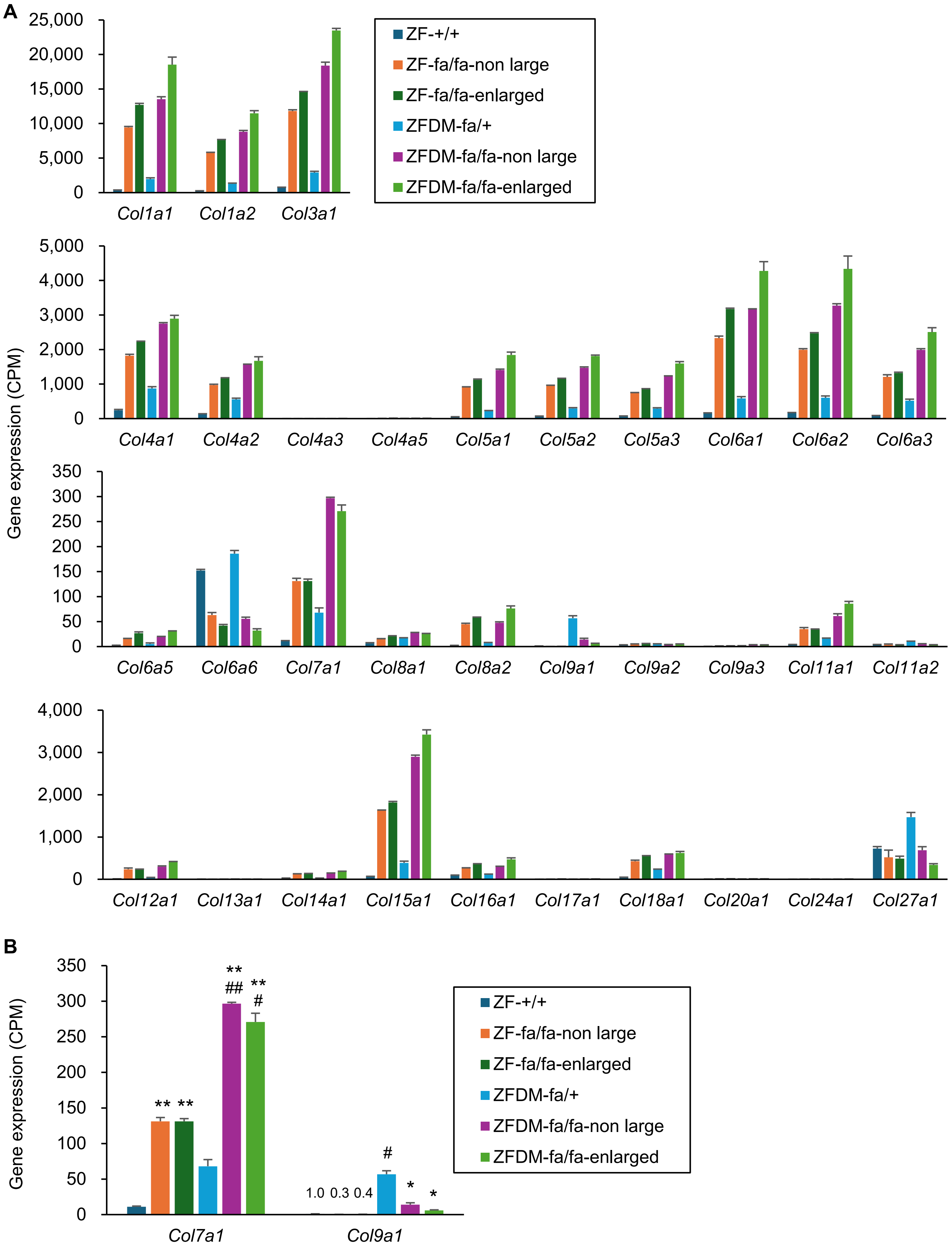

Supplementary Figure S3. Gene expression profiles of collagens in ZF and ZFDM rats at 12 weeks of age. (*A*) Expression levels of collagens in ZF and ZFDM rats at 12 weeks of age. The data are CPM values derived from the RNA-sequencing analysis and expressed as means ± SEM (n=3 each). (*B*) Expression levels of *Col7a1* and *Col9a1* in ZF and ZFDM rats at 12 weeks of age. The data are CPM values derived from the RNA-sequencing analysis and expressed as means ± SEM (n=3 each). Tukey-Kramer method was used for evaluation of statistical significance: **P* < 0.05, ***P* < 0.01 (vs. +/+ or *fa*/+ of each strain); #*P* < 0.05, ##*P* < 0.01 (vs. the corresponding group of ZF rats).
